# Optimization of the Production of Vaccine Epitopes from *Clostridium novyi* Alpha-Toxin Using Strains of Recombinant *Escherichia coli*

**DOI:** 10.3390/microorganisms13071481

**Published:** 2025-06-26

**Authors:** Mellanie K. C. Félix, Tullio T. Deusdará, Hélio S. Brito, Gil R. Santos, Eduardo R. T. Leite, Vanessa M. Chapla, Kelvinson F. Viana, Igor V. Brandi, Maria Edilene M. de Almeida, Luis André M. Mariúba, Paulo A. Nogueira, Elizângela F. da Silva, Juliane C. Glória, Raquel Stefanni R. da Silva, Darleide dos S. Braga, Anderson M. de Lima, Andreimar M. Soares, Alex Sander R. Cangussu

**Affiliations:** 1 Graduate Program for in Biodiversity and Biotechnology of Legal Amazon, Federal University of Tocantins, Palmas 77001-090, TO, Brazil; 2 Bioprocess Engineering and Biotechnology, Federal University of Tocantins, Gurupi 77410-530, TO, Brazil; 3 Graduate Program for in Environmental Chemistry, University of Tocantins, Gurupi 77410-530, TO, Brazil; 4 Interdisciplinary Center for Life Sciences and Nature, Federal University of Latin American Integration, UNILA, Foz do Iguaçu 85866-000, PR, Brazil; 5 Institute of Agricultural Sciences, Federal University of Minas Gerais, Montes Claros 39404-547, MG, Brazil; 6 PostGraduate Program in Biotechnology, UNIMONTES Campus Universitário Prof. Darcy Ribeiro, Av. Prof. Rui Braga, s/n, Vila Mauriceia, Montes Claros 39401-089, MG, Brazil; 7 Instituto Leônidas e Maria Deane, Oswaldo Cruz Foundation-Fiocruz Amazônia, Manaus 69057-070, AM, Brazil; 8 Graduate Program for in Sciences Applied to Hematology PPGH, University of Amazonas (UEA), Manaus 69050-010, AM, Brazil; 9 Graduate Program for in Biology of Host-Pathogen Interaction, Instituto Leônidas e Maria Deane, Oswaldo Cruz Foundation-Fiocruz Amazônia, Manaus 69057-070, AM, Brazil; 10 Graduate Program for in Basic and Applied Immunology, Federal University of Amazonas, Manaus 69057-070, AM, Brazil; 11 Graduate Program for in Biotechnology, Federal University of Amazonas, Manaus 69057-070, AM, Brazil; 12 INCT-CONEXAO BIO3TOX—National Institute of Science and Technology for Research and Knowledge Excellence of the Western/Eastern Amazon in Biodiversity, Biotechnology, Biometeorology and Toxicology Applied to One Health, Fiocruz Rondônia, Porto Velho 76812-245, RO, Brazil; 13 Laboratory of Biotechnology of Proteins and Bioactive Compounds, LABIOPROT, Fiocruz Rondônia, Porto Velho 76812-245, RO, Brazil

**Keywords:** alpha-toxin, vaccine epitopes, *C. novyi*, clostridiosis, bioprocesses, DE3

## Abstract

*Clostridium novyi* is a common pathogen in domestic animals and humans, and alpha-toxin is the main cause of its pathogenesis. Because it is a fastidious organism, obtaining alpha-toxin is expensive. Therefore, we proposed an in silico study to synthesize epitopes in cultures of Escherichia coli BL21 pLysS (DE3). First, we used a stirred-tank bioreactor, developing a dry mass yield (DMY) of 0.77 g/L in batch cultures and 1.03 g/L in fed-batch cultures, without acetic acid production. With scale-up using a system without mechanical agitation, there was a higher DMY (1.20 g/L) with 0.56 mmol/mL of alpha-toxin epitope 1 (DE3/Ep1) and 0.61 mmol/mL of alpha-toxin epitope 2 (DE3/Ep2), with a similar profile for O2 consumption, glucose, and no acetic acid production. The kinetic parameters µ(h^−1^), YX/S, YP/S, QP, and QX did not differ significantly; however, the kinetic data were superior. Our results suggest that in silico tools allow epitope selection and bioprocess standardization. This system provides cost savings and technological advances for the veterinary vaccine industry.

## 1. Introduction

Clostridioses are caused by bacteria of the genus *Clostridium*, which are anaerobic, mobile rods that cause significant losses in cattle farming [1,2,3,4]. *Clostridium* causes diseases mediated by toxins or tissue invasion, differing according to the expression and synthesis of genes [5,6,7,8,9]. *C. novyi* is a common agent in domestic animals and humans, classified according to toxin production. Alpha-toxin is the primary cause of pathogenesis; it alters vascular permeability, leading to necrosis and death [5,10,11]. Because *C. novyi* is a fastidious microorganism, obtaining alpha-toxin is expensive, leading to failure during various bioprocessing stages [10,12,13].

Technological alternatives such as expressing recombinant proteins using DE3 have been studied in silico to select regions corresponding to vaccine epitopes [2,14,15]. In silico studies, hydrophobicity, accessibility, antigenicity, and protein flexibility analyses are employed using GenBank data. These tools enable selectable epitopes to be acquired in constructing an expression vector [16,17,18] and are validated after synthesis in DE3 cell culture and reactivity with serum from infected animals [12,19,20,21,22,23].

Given the relevance of these immunogens in anti-*Clostridium* vaccines, we proposed optimizing and scaling vaccine epitopes from *C. novyi* alpha-toxin in DE3 culture. Here, we used a non-agitated tank biological reactor to promote improved conditions for bioprocesses and alternatives for the production of immunogens, which will enable competitive gains in the veterinary vaccine industry.

## 2. Materials and Methods

### 2.1. Bioinformatics Tools and Vaccine Epitope Expression Vector Construction

Computer programs were used to select epitopes of alpha-toxin from *C. novyi* (alpha-toxin *Clostridium novyi*–GenBank: CAA88565.1). The regions were predicted using the Immune Epitope Database Analysis Resource (IEDB-AR) (http://tools.immuneepitope.org/main, accessed on 16 January 2022). We selected two alpha-toxin epitopes from regions between amino acids 1 and 583 of the N-terminal (DE3/Ep1) and regions between amino acids 1599 and 2178 of the C-terminal region (DE3/Ep2), both obtained considering the relationship of antigenicity, flexibility, and ability to stimulate the adaptive immune response. DE3/Ep1 and DE3/Ep2 genes were synthesized by Invitrogen (Thermo Fisher Scientific, Waltham, MA, USA), modified for codon bias for Escherichia coli. These genes were inserted into the pRSET expression plasmid, which allows for the addition of a polyhistidine tail (6xHIS) at the amino terminus of each protein to facilitate purification after cell lysis. Following the manufacturer’s manual, the synthetic gene plasmid was transformed into electrocompetent Escherichia coli (DE3) pLysS using an electroporator. Then, the cells were plated in Luria–Bertani culture medium containing ampicillin (100 µg/mL) and chloramphenicol (11.4 µg/mL) and incubated overnight at 37 °C. The expression of epitopes in DE3 was performed with the induction of isopropyl-β-D-thiogalactopyranoside (IPTG) (Invitrogen^®^, Carlsbad, CA, USA) in Luria–Bertani medium with ampicillin and chloramphenicol. The cultures were performed in the first few hours until OD600 (0.6) growth at 37 °C. Induction with 1 mM IPTG was followed by temperature adjustment to 28 °C. Samples from DE3 cultures were treated with denaturing buffer (glycerol 20% (*v*/*v*), sodium dodecyl sulfate (SDS) 4% (*w*/*v*), Tris pH 6.8 (100 Mm), bromophenol blue 0.2% (*w*/*v*), beta-mercaptoethanol (200 mM), and subsequent boiling for 10 min. Polyacrylamide gel electrophoresis (SDS-PAGE 12%) was performed to validate the expression of the corresponding epitopes of de3/ep1 and de3/ep2 genes, followed by Western blotting. The design and expression of de3/ep1 and de3/ep2 in DE3 were performed in partnership with the Laboratory of Diagnosis and Control and Infectious Diseases of the Amazon, Instituto Leônidas & Maria Deane (Fiocruz Amazônia, Manaus, Brazil).

### 2.2. DE3 Cultivation on a Rotary Shaker

DE3 isolated colonies were inoculated in a semi-defined culture medium (SD) plus 100 μg/mL ampicillin in Erlenmeyer flasks of 250 mL with 50 mL culture volumes. The SD medium consisted of 5.0 g/L glucose, yeast extract 5.0 g/L, dibasic potassium phosphate (K_2_HPO_4_) 9.4 g/L, monobasic potassium phosphate (KH_2_PO_4_) 5.0 g/L, sodium chloride (NaCl) 5.0 g/L, zinc chelate (EDTA-Zn) 1.0 g/L (Comnagro^®^, Campinas, Brazil), and manganese chelate (EDTA-Mn) 0.2 g/L (Comnagro^®^). The studies were carried out in triplicate on an SL-223/F rotary shaker (Solab Cientifica^®^, Piracicaba, SP, Brazil) at 37 °C, at 200 rpm for 16 h. Inoculum fractions corresponding to 2.5%, 5%, and 10% were evaluated with sampling, optical density readings (OD600), and the measurement of specific growth rate (µh^−1^) and DMY.

### 2.3. DE3 Cultivation in SD Medium in a Stirred-Tank Biological Reactor

Batch and fed-batch cultures were created in a Tecnal BIO–TEC^®^ stirred-tank biological reactor with a capacity of 1 L, consisting of a jacketed glass container, digital control unit, and pH 7 control using 3M sodium hydroxide (NaOH) at 37 °C. We used a dissolved oxygen supplementation strategy with cascade control of agitation from 100 to 1000 rpm, with specific air supply flow input fixed at 3 vvm. Inoculum propagation was performed initially from the stock culture of each DE3 (1.5 mL) activated in 8.5 mL of SD medium, under agitation at 200 rpm for 12 h at 37 °C while maintaining an inoculum fraction of 10% in the bioreactor. During the process in a fed-batch culture, 7 mL of glucose solution 500 g/L containing ampicillin at a concentration of 100 μg/mL was added every 30 min until reaching a stationary growth phase. Samples were collected at 1 h intervals to determine growth kinetics, acetate and glucose consumption, and the synthesis of DE3/Ep1 and DE3/Ep2.

### 2.4. Scaling Up DE3 Cultivation in SD Medium in an Unstirred-Tank Biological Reactor

The fed-batch cultures were carried out in a biological reactor with a non-agitated tank with a capacity of 20 L, consisting of an adapted vessel fitted with a wide-mouth round carboy (Thermo Scientific Nalgene^®^, Rochester, NY, USA) with low-density polyethylene handles to facilitate transport and dispensing. The receptacle was fitted with a screw cap (83B) of white polypropylene. The cultures were carried out at pH 7 control using 3 M NaOH at 37 °C. The dissolved oxygen supplementation strategy involved a fixed air inlet at 3 vvm, without mechanical agitation. Inoculum propagation was performed using a stock culture of each DE3 (1.5 mL) activated in 8.5 mL of SD medium with agitation at 200 rpm for 12 h at 37 °C. We maintained an inoculum fraction of 10% for cultivation in the bioreactor with a working volume of 10 L. During the fed-batch process, 100 mL of 500 g/L glucose solution and 100 μg/mL ampicillin were added at 30 min intervals until a stationary growth phase was reached. Samples were collected at 1 h intervals to determine growth kinetics, acetate and glucose consumption, and the synthesis of DE3/Ep1 and DE3/Ep2.

### 2.5. System for Induction and Purification of DE3/Ep1 and DE3/Ep2 Recombinant Epitopes

In the first induction stage, we used three 35 mL aliquots of 10 g/L lactose solution in the cultures grown in the biological reactor of a stirred tank, soon after reaching maximum cell growth, followed by another addition after two subsequent hours of cultivation. In the scale-up using a non-agitated tank biological reactor, we used three 500 mL aliquots of 10 g/L lactose with 0.2 mM of IPTG at the same time intervals as the laboratory-scale experiments. All induction studies were performed for 6 h at 28 °C.

A chromatography system (Akta Purifier 10^®^, GE Lifescience Healthcare^®^, Singapore) was used for the DE3/Ep1 and DE3/Ep2 purification steps. The collected fractions were lyophilized and stored at −20 °C for analysis. Reverse-phase chromatography was performed as proposed [24]. The DE3/Ep1 and DE3/Ep2 fractions from affinity chromatography after lyophilization was solubilized in 0.1% trifluoroacetic acid (TFA) (solution A) (Merck^®^, Darmstadt, Germany) and subjected to high-performance liquid chromatography in a C-18 column (25 mm × 4.6 mm, Supelco^®^, Bellefonte, PA, USA), previously equilibrated with solution A and eluted under a gradient from 0 to 70% with solution B (99.9% acetonitrile (Merck^®^) and 0 TFA, 1%) in five column volumes under a flow rate of 1 mL/min. Elutions were monitored at 280 nm. Profiles referring to DE3/Ep1 and DE3/Ep2 were obtained, confirming the degree of purification and product concentration. The collected fractions were visualized again on 12% SDS-PAGE to check the purity and confirm antigenicity using Western blotting.

### 2.6. Western Blotting

Bands referring to DE3/Ep1 and DE3/Ep2 separated on 12% SDS-PAGE gels were transferred to nitrocellulose membranes using the BioRad^®^ (Hercules, CA, USA) protocol. A primary antibody from the serum of Swiss mice infected with wild-type *C. novyi* type B alpha-toxin was used. In the negative control, serum from healthy Swiss mice was used. Secondary anti-mouse antibody IgG-rabbit A9044 (Sigma^®^, St. Louis, MO, USA) (conjugated with peroxidase at 1:2000 and 0.05% (*v*/*v*) of 3,3′-diaminobenzidine) was used to certify the reactivity with purified DE3/Ep1 and DE3/Ep2, compared to inactivated wild-type alpha-toxin.

### 2.7. Fermentation Parameters

#### 2.7.1. Determination of Glucose Consumption

The determination of glucose consumption was performed using the liquid enzymatic glucose system (Labtest^®^, Lagoa Santa, MG, Brazil), Equation (1), where the calculation is composed of two reactions using glucose oxidase (GOD) and peroxidase (POD), according to Bergmeyer [25].

(1) $$Glucose+O_{2}+H_{2}O\;\;(\underset{\to }{GOD)\;}Gluconicacid+H_{2}O_{2}\;2H_{2}O_{2}+4-aminoantipyrine+phenol\;\;\underset{\to }{(POD)\;\;\;}antipyrylquinonimine+4H_{2}O$$

The product formed (4-antipyrliquinonimine) has a reddish color, and its intensity is directly proportional to the glucose concentration. The assays were prepared in 2 mL shaker tubes, incubated for 5 min at 37 °C, and the reading was performed at 510 nm. The glucose concentration was calculated as described in Equation (2).

(2) $$Glucose=\frac{TestAbs}{Std.Abs}\cdot \;100\;(\mathrm{mg}/\mathrm{dL})$$

#### 2.7.2. Determination of Acetic Acid (Hac) and DMY Production

The determination of HAc was performed using a Shimadzu^®^ chromatograph (SPD–M2OA), equipped with a high-precision LC-6AD pump (CBM-20A) and ultraviolet detectors that detect in the absorbance range of 190 to 700 nm. The ion exclusion column used was the C-18 Shim-pack^®^ column (250 × 4.6 mm, Shimadzu^®^). The analysis was conducted for 12 min at 30 °C with a mobile phase flow rate of 0.6 mL/min. The mobile phase consisted of a 1% phosphoric acid (H_3_PO_4_) solution and an injection volume of 20 µL. The samples were previously filtered using a C-18 Chromabond^®^ membrane (3 mL/500 mg), and readings were taken at 204 nm. The determination of HAc was performed using a standard curve and linear regression.

The determination of DMY was calculated as the difference between mass A (mA) and mass B (mB), where mA is the mass of the crucibles without sample, mB is the mass of the crucibles containing samples from the culture, and Al is the volume of the sample. DMY values were generated by correlating the dry weights and OD600 of samples from the culture, with one (1) unit of OD600 corresponding to 0.61 g/L of dry cell weight. The dry mass correlation was determined as described in Equation (3).

(3) $$DMY=\frac{\left(mA-mB\right)}{Al}\cdot 1000\left(g/L\right)$$

#### 2.7.3. Yield Coefficients

The specific growth rate (μ) was determined during the exponential phase of bacterial growth, in which the specific growth rate is constant and maximum (µ_x = µ_m). μ values were obtained through Equation (4), where dX is the difference in OD600 at a specific time point and a subsequent time point, dt is the time variation, and X is the concentration of cells at that specific time point.

(4) $$\mu =(\frac{dX}{dt})/X\;(h^{-1})$$

The yield coefficients (y) were analyzed in terms of biomass ($Y_{\frac{X}{S}}$) and product ($Y_{\frac{P}{S}}$), visualized in Equations (5) and (6). To calculate the biomass-to-product conversion factor, cell growth (X) and substrate consumption (S) were considered, as shown in Equation (5). To calculate the product yield, the amount of product (P) and the substrate consumption (S) were considered, as described in Equations (6)–(8), which define the volumetric productivity of the product $Q_{P}$ and of the biomass $Q_{X}$ in relation to time (t).

(5) $$Y_{\frac{X}{S}}=\frac{X_{f}-X_{i}}{S_{i}-S_{f}}\;(g/\mathrm{mmol}.\mathrm{mL})$$

(6) $$Y_{\frac{P}{S}}=\frac{P_{f}-P_{i}}{S_{i}-S_{f}}\;(\mathrm{mmol}/\mathrm{mL}\cdot {\left(\mathrm{mmol}/\mathrm{mL}\right)}^{-1})$$

(7) $$Q_{P}=\frac{P_{f}-P_{i}}{t}\;(\mathrm{mmoL}./\mathrm{mL}\cdot h)$$

(8) $$Q_{X}=\frac{X_{f}-X_{i}}{t}\;(g/\mathrm{mL}\cdot h)$$

### 2.8. Statistical Analysis

The experiments were carried out following a completely randomized design employing analysis of variance. Kinetic parameters obtained in stirred and non-agitated systems were comparatively analyzed. Data means were analyzed using Student’s *t*-test, considering *p* < 0.05 as significant using Origin Pro^®^ 8.5 software.

## 3. Results

### 3.1. Detection of Vaccine Epitopes

**Figure 1:**
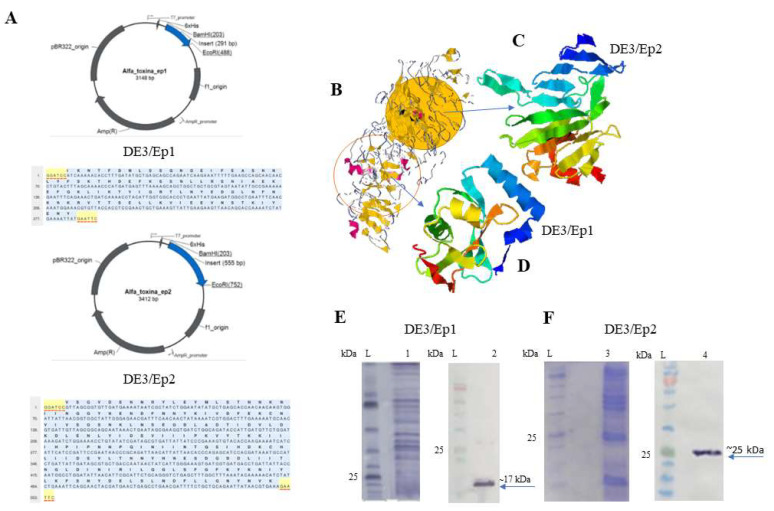
Sequence encodes *C. novyi* type B alpha-toxin epitopes, silico modeling, and detection. (**A**) Plasmid design and sequences; (**B**) 3D structure of *C. novyi* type B alpha-toxin fraction predicted by i-TASSER [26] from GenBank data: CAA88565.1 [27]; (**C**,**D**) 3D structures of DE3/Ep1 and DE3/Ep2, respectively. (**E**,**F**) Detection of DE3/Ep1 (17 kDa) and DE3/Ep2 (26 kDa) using 12% gel electrophoresis and Western blotting of vaccine epitope reactivity with serum from Swiss mice infected with wild-type *C. novyi* alpha-toxin.

Protein regions of the predicted and selected *C. novyi* alpha-toxin revealed the potential for the selection of epitopes, and the region corresponding to DE3/Ep1 is located in the N-terminal domain, with a hydrophobic domain that mediates the translocation of the toxin to the cytosol, while DE3/Ep2 is constituted in the C-terminal domain (also characterized as a hydrophobic domain). In silico and DE3-expressed models revealed bands of 17 kDa and 26 kDa corresponding to DE3/Ep1 and DE3/Ep2, respectively (Figure 1). Reactivity with serum from mice infected with wild-type *C. novyi* alpha-toxin confirmed that these selected regions are recognized by specific antibodies present in the context of infection (Figure 1).

### 3.2. Optimization and Scaling of Vaccine Epitope Production

**Figure 2:**
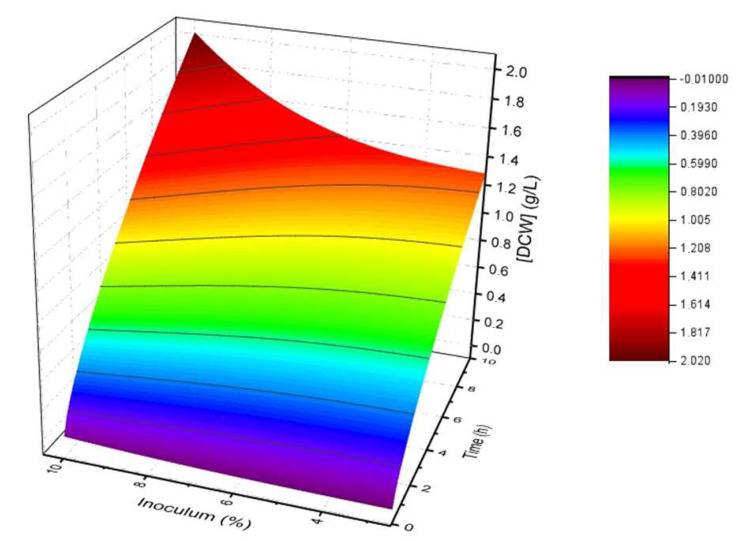
DE3 cultivation response surface on a rotary shaker and DMY ratio, inoculum fraction as a function of cultivation time (h) using response equations, and analysis of variances using Origin Pro^®^ 8.5 software. Data are represented as means and standard deviations, considering *p* < 0.05.

**Figure 3:**
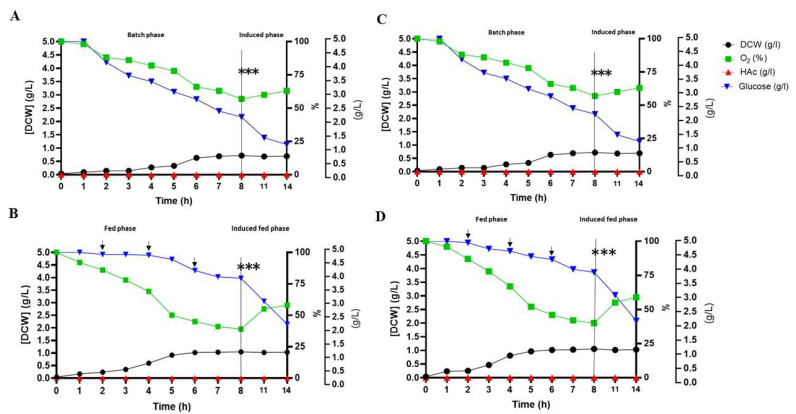
Optimization of vaccine epitope production in a stirred-tank biological bioreactor. (**A**,**B**): DE3 kinetic profiles targeting the single-batch synthesis of DE3/Ep1 and DE3/Ep2 epitopes. (**C**,**D**): kinetic profile of DE3 targeting synthesis of DE3/Ep1 and DE3/Ep2 in the fed-batch process. Data obtained from DE3 cultures in an SD medium containing ampicillin (100 µg/mL) was conducted in a benchtop bioreactor (Tecnal BIO–TEC^®^) at 37 °C. Glucose supplementation (500 g/L) occurred at 30 min intervals until maximum DMY. Induction was conducted at 28 °C for 6 h using lactose (10 g/L) as an inducer. DMY—dry mass yield (g/L), O_2_—dissolved oxygen (%), HAc—acetic acid (g/L), and glucose (g/L). (***) start of induction, (→) start of feeding. Data are represented as means and standard deviations, considering *p* < 0.05 using Origin Pro^®^ 8.5 software.

**Figure 4:**
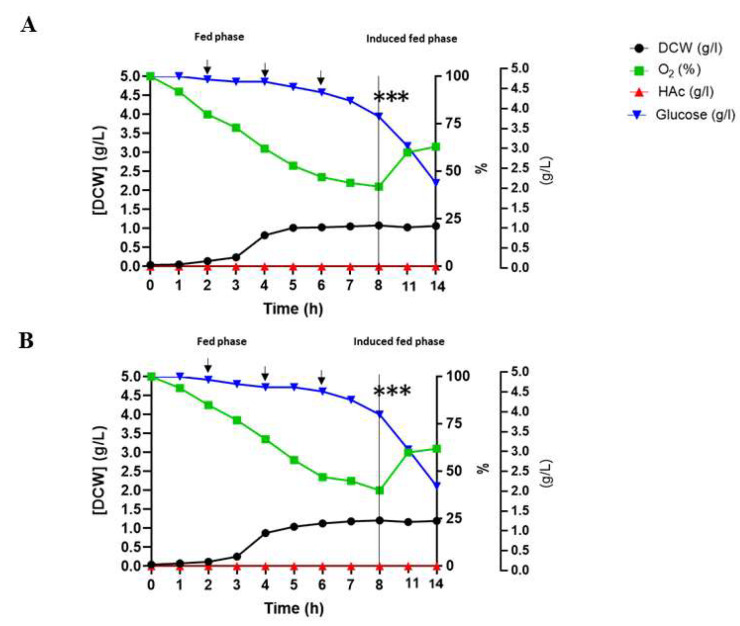
Scheduling the production of vaccine epitopes in a non-stirred-tank biological bioreactor. (**A**,**B**) Kinetic profiles of DE3 targeting synthesis of DE3/Ep1 and DE3/Ep2 epitopes. Data obtained from DE3 cultures in SD medium containing ampicillin (100 µg/mL), conducted in a Tecnal BIO–TEC^®^ bioreactor with an adapted vessel using round Thermo Scientific Nalgene^®^ carboys at 37 °C. Glucose supplementation (500 g/L) occurred at 30 min intervals until maximum DMY. Induction was conducted at 28 °C for 6 h using a combination of lactose (10 g/L) and IPTG (0.2 mM) as inducers. DMY—dry mass yield (g/L), O_2_—dissolved oxygen (%), HAc—acetic acid (g/L), and glucose (g/L). (***) start of induction, (→) start of feeding. Data are represented as means and standard deviations, considering *p*<0.05 using Origin Pro^®^ 8.5 software.

Response surface modeling data showed that the 10% inoculum fraction (*v*/*v*) allowed for a greater DMY and µ(h^−1^) (Figure 2). The optimization of the production of epitopes in DE3 at the laboratory scale revealed a maximum yield of 0.77 g/L of DMY, with an adequate supply of O2, the total depletion of glucose, and no significant production of HAc (Figure 3A,C). The µ(h^−1^), YX/S, YP/S, QP, and QX did not differ significantly from one another during the production of DE3/Ep1 and DE3/Ep2 (Table 1). In the fed-batch culture under the same induction conditions, the yield was higher (1.03 g/L), with no evidence of HAc production (Figure 3B,D). For the scaling of DE3, the maximum yield of DMY was 1.20 g/L, showing no difference in the production of epitopes, O2 consumption, glucose, or production of HAc (Figure 4A,B, Table 2).

### 3.3. DE3 Induction Strategy and Vaccine Epitope Purification

**Figure 5:**
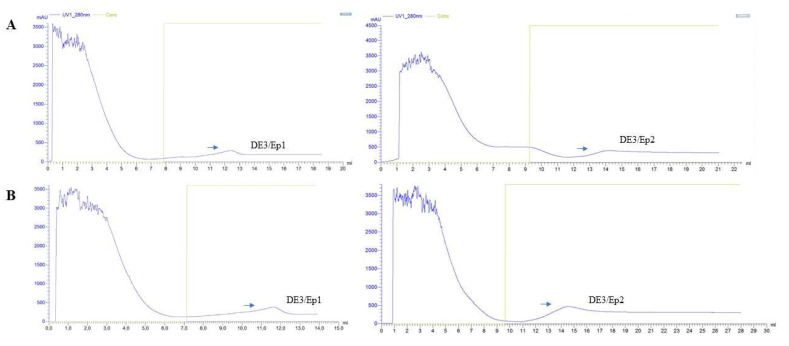
Purification of vaccine epitopes in a lab-scale stirred-tank biological bioreactor. (**A**) A single batch obtained profiles of purified DE3/Ep1 and DE3/Ep2 epitopes. (**B**) Profiles of purified DE3/Ep1 and DE3/Ep2 epitopes were obtained in the fed-batch culture. Data obtained in the Akta Purifier 10^®^ chromatography system, GE Lifescience Healthcare^®^. Eluates were determined at 280 nm.

**Figure 6:**
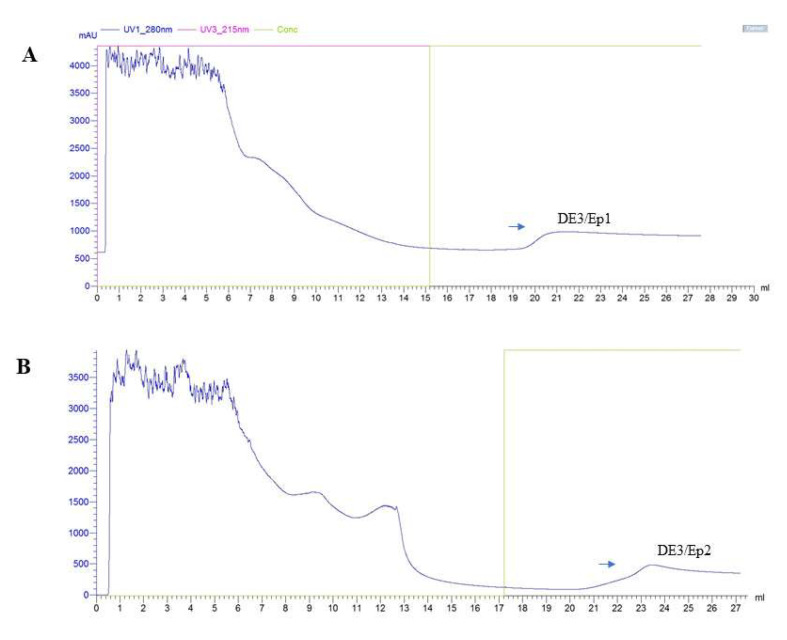
Purification of vaccine epitopes in a scale-up non-stirred-tank biological bioreactor. (**A**,**B**): profiles of purified DE3/Ep1 and DE3/Ep2 epitopes obtained by the fed-batch culture. Data obtained in the Akta Purifier 10^®^ chromatography system, GE Lifescience Healthcare^®^. Eluates were determined at 280 nm.

The induction strategy using lactose aliquots revealed DE3/Ep1 and DE3/Ep2 production profiles in both processes (batch and fed-batch) conducted in a 1 L stirred-tank biological reactor. Peak production of DE3/Ep1 and DE3/Ep2 was recorded in the ranges from 300 mAU (single-batch) to 400 mAU (fed-batch), with higher production in the fed-batch system (Figure 5A,B). Scaling up the induction of DE3 (with lactose and IPTG) revealed higher production peaks of purified epitopes with 1000 mAU and 600 mAU (Figure 6A,B). The combination of lactose and IPTG showed promise and can be explored by evaluating different combinations in future scaling studies to induce recombinant protein in DE3 cultures. Purified epitopes showed concentrations corresponding to 0.56 mmol/mL of DE3/Ep1 and 0.61 mmol/mL of DE3/Ep2 (Figure 1, Table 2).

## 4. Discussion

*C. novyi* type B is a fastidious microorganism critical in cattle breeding because it causes hepatic necrosis in ruminants and carries a high mortality rate, resulting in severe economic losses [2,8,10,11]. Commercial multi-antigen vaccines require inactivated alpha-toxin of *C. novyi* type B as an immunogen for herd protection. Technological alternatives such as expressing recombinant proteins using DE3 bacterial cells have been the primary target of studies [2,10,12,13,28,29,30]. We applied this technological strategy using in silico tools, analyzing the hydrophobicity, accessibility, antigenicity, and flexibility of alpha-toxin data from *C. novyi* (GenBank: CAA88565.1). We selected DE3/Ep1 and DE3/Ep2 epitopes, which later proved reactive with serum from animals infected with wild-type active alpha-toxin of *C. novyi* (Figure 1A,B). Previous in silico studies revealed similar data, confirming the antigenicity and immunogenicity of selectable epitopes expressed in DE3 cell cultures [19,20,21].

Recombinant antigens have become a promising alternative [20,31]. Zeng [32] proposed the development of a recombinant vaccine using antigen expression in DE3 and obtaining the epitope of *Clostridium perfringens* beta toxin using an in silico tool for the selection and confirmation of antibody production [29]. To optimize the production of DE3/Ep1 and DE3/Ep2 epitopes in DE3, we initially applied a response surface analysis (RSA) and determination of the inoculum fraction of DE3 [33]. We obtained the highest cell concentration and shorter bioprocessing times when an inoculum fraction of 10% (*v*/*v*) DE3 was used (Figure 2). The RSA provided screening of the determining parameters for obtaining bioproducts through the polynomial model, allowing for the directed optimization of data [34]. It is usually used as a statistical tool to evaluate control parameters and their interactions, saving experiments and reducing time and resources needed to improve processing conditions in bioreactors [35,36,37].

Our DE3 kinetics data and DE3/Ep1 and DE3/Ep2 epitope yield coefficients revealed higher yields in the fed-batch process. The addition of substrate during the bioprocess allows an increase in cell concentration to the detriment of the production of HAc, which is considered the primary DE3 inhibitor [38]. With the addition of glucose, the inactivation of catabolite-activating proteins occurs, which act in the synthesis of enzymes, the direction for synthesis and microbial growth, greater gene expression, and (consequently) greater synthesis of the product of interest [39]. HAc is a product of DE3 fermentation metabolism generated by pyruvate oxidation via acetyl coenzyme A during the exponential phase, in which glucose is not limiting [40]. At high glucose concentrations, intracellular levels of cyclic adenosine 3′,5′-monophosphate are low, and it does not activate the expression of acetyl coenzyme A synthetase, resulting in the accumulation of HAc, growth inhibition, and the synthesis of bioproducts (DE3/Ep1 and DE3/Ep2) [38,40,41]. Kilikian [42] observed that in DE3 cultures, an HAc concentration of up to 0.9 g/L allows the development of DMY. Suárez and Kilikian [43] observed that µ(h^−1^) (0, 3) might be the growth velocity that favors the accumulation of HAc. We did not identify an inhibitory concentration of HAc in fed-batch DE3 cultures, revealing that the feeding and induction strategies were efficient (Figure 3 and Figure 4).

We found that DE3 induction, using the association of IPTG and lactose, can be an efficient and cost-effective strategy. Tian [44] compared induction with concentrations of lactose ranging from 2 to 50 g/L and found that 10 g/L provided the most significant activity of bioproducts. Bashir [45] determined the effect of induction, revealing efficient lactose concentrations at 14 g/L. Lactose can be used as an inducer in processes using DE3, which are advantageous because of their natural origin, lower cost, lower toxicity, and low speed of induction [46,47]. On the other hand, IPTG has been more at 0.1 to 1 mM, with higher concentrations being toxic [46,47,48,49,50]. The association of lactose and IPTG may represent an efficient and lower-cost strategy that can be explored in future studies; there are still limited data on inducing DE3 in the synthesis of recombinant bioproducts [46,47,50].

Recombinant epitopes are commonly purified in Akta Purifier systems [26,51,52]. Zeng [32] and Yu [53] used the Akta Purifier system to purify toxins and recombinant epitopes of *Clostridium* obtained in DE3. Our purification data revealed the separation profile and characteristic peaks of DE3/Ep1 and DE3/Ep2 obtained in the fed-batch process using a system without mechanical agitation that allows for a reduction in operational costs and energy consumption and provides technological advances for obtaining immunogens.

Several studies have already been conducted to elucidate the protein structures, using a hierarchical approach for protein structure and function prediction [53,54,55]. Here, we employed the database alpha-toxin *Clostridium novyi*–GenBank: CAA88565.1, predicted using the Immune Epitope Database Analysis Resource (IEDB-AR) (http://tools.immuneepitope.org/main, accessed on 16 January 2022), the I-TASSER server that revealed data about hydrophobicity, accessibility, antigenicity, and protein flexibility and employed for the selection of epitopes. Despite the relevance of the data obtained in selecting vaccine epitopes using a bioinformatics tool from *C. novyi* alpha-toxin data and the proposal to employ alternative bioreactors in a non-stirred system, questions remain that must be explored.

Advances in studies that portray metabolic pathways and intermolecular interactions of DE3/Ep1 and DE3/Ep2 are relevant, as are studies that reveal optimal proportions of lactose and IPTG used for the induction of DE3. Our findings confirmed a promising strategy for obtaining vaccine epitopes related to immunogens of economic relevance, suggesting new industrial production routes that can be used to immunize herds against clostridioses. In summary, our results suggest that in silico tools allow epitope selection and bioprocess standardization, which provide cost savings and technological advances for the veterinary vaccine industry.

## Figures and Tables

**Table 1:** Fermentative parameters of DE3 cultures in a laboratory-scale stirred-tank biological bioreactor. Specific formation rate, *µX* (h^−1^); volumetric productivity, *Q_P_* (mmol·L^−1^·h^−1^); yields coefficients: *Y_E/S_* (mmol·L^−1^ (mmol·L^−1^)^−1^), *Y_G/S_* (mmol·L^−1^ (mmol/mL·h), *Y_P/S_* (mmol/mL·(mmol/mL)^−1^), *Y_X/S_* (g/mmol·mL).

Gene Name	Sequence Type	Express Cloning Vector	System	Cell	Bioreactor	Induction	Yield Coefficient					
							*µ* (h^−1^)	*P* (mmol/mL)	*Y_X/S_* (g/mmol·mL)	*Y_P/S_* ((mmol/mL)(mmol/mL)^−1^)	*Q_P_* (mmol/mL·h)	*Q_X_* (g/mL·h)
Alpha-toxin (de3/Ep1)	Protein	pRSET A	batch	DE3	Lab-scale 1 L	Lactose	0.24	0.56	30.46	0.26	0.03	8.49
			fed-batch	DE3	Lab-scale 1 L	Lactose	0.28	0.56	62.07	0.35	0.03	12.49
Alpha-toxin (de3/Ep2)	Protein	pRSET A	batch	DE3	Lab-scale 1 L	Lactose	0.25	0.61	34.35	0.29	0.03	9.15
			fed-batch	DE3	Lab-scale 1 L	Lactose	0.29	0.61	61.28	0.37	0.04	12.73

**Table 2:** Fermentative parameters of DE3 cultures in a scale-up non-stirred-tank biological bioreactor. Specific formation rate, *µX* (h^−1^); volumetric productivity, *Q_P_* (mmol·L^−1^·h^−1^); yields coefficients: *Y_E/S_* (mmol·L^−1^ (mmol·L^−1^)^−1^), *Y_G/S_* (mmol·L^−1^ (mmol/mL·h), *Y_P/S_* (mmol/mL·(mmol/mL)^−1^), *Y_X/S_* (g/mmol·mL).

Gene Name	Sequence Type	Express Cloning Vector	System	Cell	Bioreactor	Induction	Yield Coefficient					
							*µ* (h^−1^)	*P* (mmol/mL)	*Y_X/S_* (g/mmol·mL)	*Y_P/S_* ((mmol/mL)(mmol/mL)^−1^)	*Q_P_* (mmol/mL·h)	*Q_X_* (g/mL·h)
Alpha-toxin (de3/Ep1)	Protein	pRSET A	fed-batch	DE3	Scale-up 20 L	Lactose/IPTG	0.30	0.56	65.76	0.36	0.04	12.97
Alpha-toxin (de3/Ep2)	Protein	pRSET A	fed-batch	DE3	Scale-up 20 L	Lactose/IPTG	0.31	0.61	71.85	0.38	0.04	14.57

## Data Availability

The original contributions presented in this study are included in the article/Supplementary Material. Further inquiries can be directed to the corresponding author.

## Supplementary Materials

The following supporting information can be downloaded at: https://www.mdpi.com/article/10.3390/microorganisms13071481/s1, Figure S1. Standard HAc chromatogram using Shimadzu^®^ (SPD–M2OA). HAc peak revealed at 204 nm with retention time 6 min. Figure S2. Chromatogram of HAc production in DE3 cultures during vaccine epitope production conducted in a lab-scale stirred-tank biological bioreactor. (A) Simple batch. (B) Batch fed. HAc peak revealed at 204 nm with a retention time of 6 min using Shimadzu^®^ (SPD–M2OA). Figure S3. Chromatogram of HAc production in DE3 cultures during vaccine epitope production conducted in a scale-up non-stirred-tank biological bioreactor. (A) DE3/Ep1. (B) DE3/Ep2. The HAc peak was revealed at 204 nm with a retention time of 6 min using Shimadzu^®^ (SPD–M2OA).

## Nomenclature

*C. novyi*	*Clostridium novyi* type B
DE3	*Escherichia coli* BL21 pLysS (DE3)
DMY	Dry mass yield (g/L)
HAc	Acetic acid (g/L)
O_2_	Dissolved oxygen (%)
µ	Specific growth rate (h^−1^)
*Y*_X/S_	Biomass yield coefficient
*Y*_P/S_	Product yield coefficient
*Q*_P_	Product volumetric productivity
*Q*_X_	Volumetric productivity of biomass
IEDB-AR	Immune Epitope Database *Analysis Resource*
DE3/Ep1	Epitope 1 of alpha-toxin transformed into DE3
DE3/Ep2	Epitope 2 of alpha-toxin transformed into DE3
de3/ep1	Alpha-toxin genetic sequence encoding DE 3/Ep1
de3/ep2	Alpha-toxin genetic sequence encoding DE 3/Ep2
pRSET	Expression plasmid
IPTG	Isopropyl-β-D-thiogalactopyranoside
SD	Semi-defined culture medium with zinc and manganese chelate
EDTA-Zn	Zinc chelate
EDTA-Mn	Manganese chelate
TFA	Trifluoroacetic acid
GOD	Glucose oxidase
POD	Peroxidase
H_3_PO_4_	Phosphoric acid
RSA	Response surface analysis
