# Supplementary material for: Optimization of the Production of Vaccine Epitopes from Clostridium novyi Alpha-Toxin Using Strains of Recombinant Escherichia coli
Source: Microorganisms. 2025 Jun 26;13(7):1481. doi: 10.3390/microorganisms13071481 (PMC12300942; doi:10.3390/microorganisms13071481)
Supplement: Supplementary file 1 [file microorganisms-13-01481-s001.zip › microorganisms-3676146-supplementary.pdf]

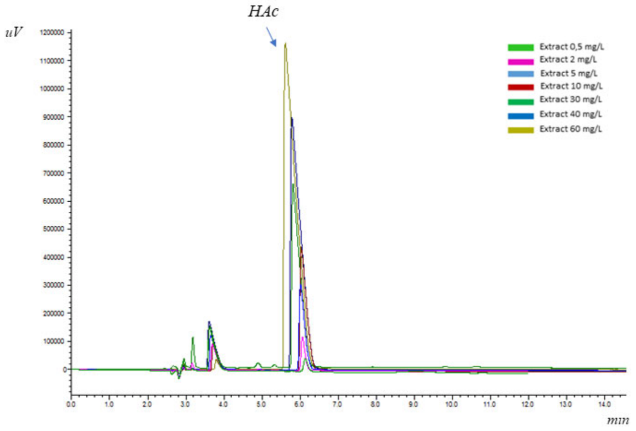

Supplementary Figure S1. Standard HAc chromatogram using Shimadzu® (SPD - M2OA). HAc peak revealed at 204 nm with retention time 6 min.

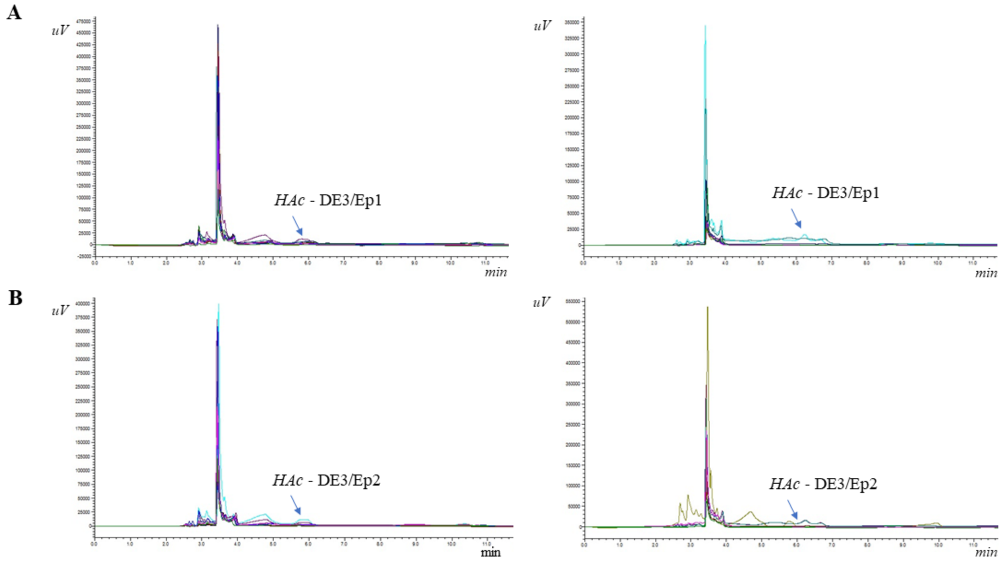

Supplementary Figure S2. Chromatogram of HAc production in DE3 cultures during vaccine epitope production conducted in a lab-scale stirred-tank biological bioreactor. (A) Simple batch. (B) Batch fed. HAc peak revealed at 204 nm with a retention time of 6 min using Shimadzu® (SPD - M2OA).

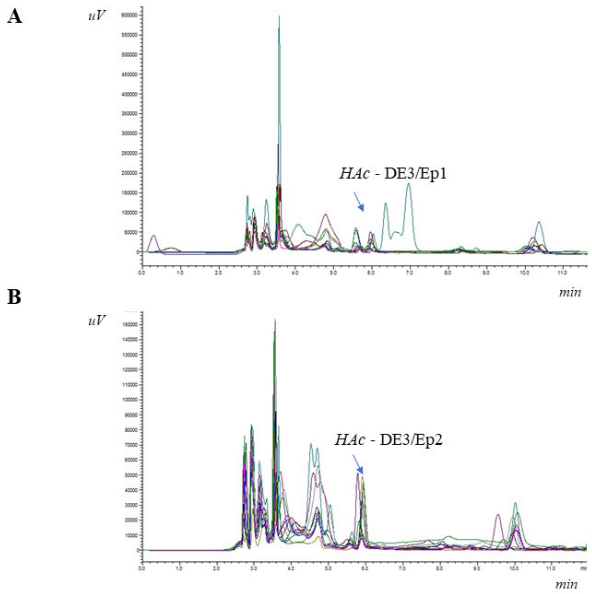

Supplementary Figure S3. Chromatogram of HAc production in DE3 cultures during vaccine epitope production conducted in a scale-up non-stirred-tank biological bioreactor. (A) DE3/Ep1. (B) DE3/Ep2. The HAc peak was revealed at 204 nm with a retention time of 6 min using Shimadzu® (SPD - M2OA).
